# Brain bases of language selection: MEG evidence from Arabic-English bilingual language production

**DOI:** 10.3389/fnhum.2015.00027

**Published:** 2015-02-05

**Authors:** Esti Blanco-Elorrieta, Liina Pylkkänen

**Affiliations:** ^1^NYUAD Institute, New York University Abu DhabiAbu Dhabi, United Arab Emirates; ^2^Department of Linguistics, New York UniversityNew York, NY, USA; ^3^Department of Psychology, New York UniversityNew York, NY, USA

**Keywords:** bilingualism, speech production, natural cue, MEG, language selection

## Abstract

Much of the world's population is bilingual, hence, language selection is a core component of language processing in a significant proportion of individuals. Though language selection has been investigated using artificial cues to language choice such as color, little is known about more ecologically valid cues. We examined with MEG the neurophysiological and behavioral effects of two natural cues: script and cultural context, hypothesizing the former to trigger more automatic language selection. Twenty Arabic-English bilinguals performed a number-naming task with a Match condition, where the cue and target language of response matched, and a Mismatch condition, with opposite instruction. The latter addressed the mechanisms responsible for overriding natural cue-language associations. Early visual responses patterned according to predictions from prior object recognition literature, while at 150–300 ms, the anterior cingulate cortex showed robust sensitivity to cue-type, with enhanced amplitudes to culture trials. In contrast, a mismatch effect for both cue-types was observed at 300–400 ms in the left inferior prefrontal cortex. Our findings provide the first characterization of the spatio-temporal profile of naturally cued language selection and demonstrate that natural but less automatic language-choice, elicited by cultural cues, does not engage the same mechanisms as the clearly unnatural language-choice of our mismatch tasks.

## Introduction

Although any message can be transmitted in a number of different ways, every time an individual speaks, a selection of specific lexical items needs to be achieved. In addition to the usual selection demands, bilingual individuals also need to choose the appropriate language in which each concept will be expressed. Bilingual language selection and switching has been widely investigated using artificial cues to language choice such as color (Meuter and Allport, 1999; Costa and Santesteban, 2004; Costa et al., 2006). However, the role of more naturalistic cues in language choice has not been characterized. This study provides an initial exploration of this uninvestigated subject. Using script and cultural context as natural cues and taking advantage of the millisecond by millisecond resolution of magnetoencephalography (MEG), we examine how naturalistic cues are processed and whether the brain mechanisms of language choice are modulated by the nature of the cue. Specifically, we aimed to test the intuition that script provides a more automatic link to language than cultural context, although both are clearly ecologically valid.

The process of selecting appropriate lexical representations encompasses two stages: concept selection (i.e., the selection of the conceptual information to be lexicalized) and lexical selection (i.e., the selection of the response word from a set of activated words) (Schriefers et al., 1990; Peterson and Savoy, 1998). Concept selection is usually a fast process that happens automatically once the communicative intention of a certain situation is clear. In contrast, successful lexical selection requires the extraction of various types of information from the environment before the most suitable word for the context can be determined. Environmental attributes relevant for this include the identity of the interlocutor, the speaker's relationship with them, the current situation, and so forth. Based on all this information, provided by available cues in the environment, the most suitable word can then be selected.

In the bilingual brain, the usual lexical selection demands are compounded by the need to select an appropriate target language. The lexicons of bilingual individuals have two words for most concepts (Kroll and Stewart, 1994; Francis, 1999; Gollan and Kroll, 2001; Garbin et al., 2010). This means that every time they speak, in addition to considering aspects such as tone or register, they also have to make a decision about which language is appropriate for the current situation. A number of studies have found that bilinguals suffer from interference and competition during the course of language production and comprehension (Abutalebi and Green, 2007; Khateb et al., 2007; Wang et al., 2007, 2009) suggesting that even if they only use one language at a time, the other language is constantly at a certain level of readiness (Grainger and Dijkstra, 1992; Grosjean, 2001). However, evidence shows that bilinguals are generally successful at selecting their target language and accessing this lexicon with very few intrusions from their other language (Poulisse and Bongaerts, 1994; Poulisse, 1999).

Several studies have investigated bilingual lexical selection both in comprehension (Beauvillain and Grainger, 1987; Schriefers et al., 1990; Dijkstra et al., 1998, 1999) and in production. The production studies have typically used numeral or picture naming as the experimental task, with some exogenous cue, typically color, indicating the desired language of response (Meuter and Allport, 1999; Costa et al., 2000, 2006; Jackson et al., 2001; Costa and Santesteban, 2004; Hoshino and Kroll, 2008; Abutalebi et al., 2011). Specifically, in these designs, one color is assigned at random to each of the languages at play, and participants produce utterances in each of the languages depending upon the color in which the item to name is presented (Meuter and Allport, 1999; Costa and Santesteban, 2004; Costa et al., 2006; Abutalebi et al., 2011). Thus, the association between a color and the language that it cued was arbitrary and had to be memorized prior to the beginning of the experiment. Importantly, no study so far has studied the processing of naturally occurring cues to language selection. The goal of the current work was to address this basic question as well as to shed light on whether the neural mechanisms of language choice are modulated by the nature of the cue.

**Figure 1:**
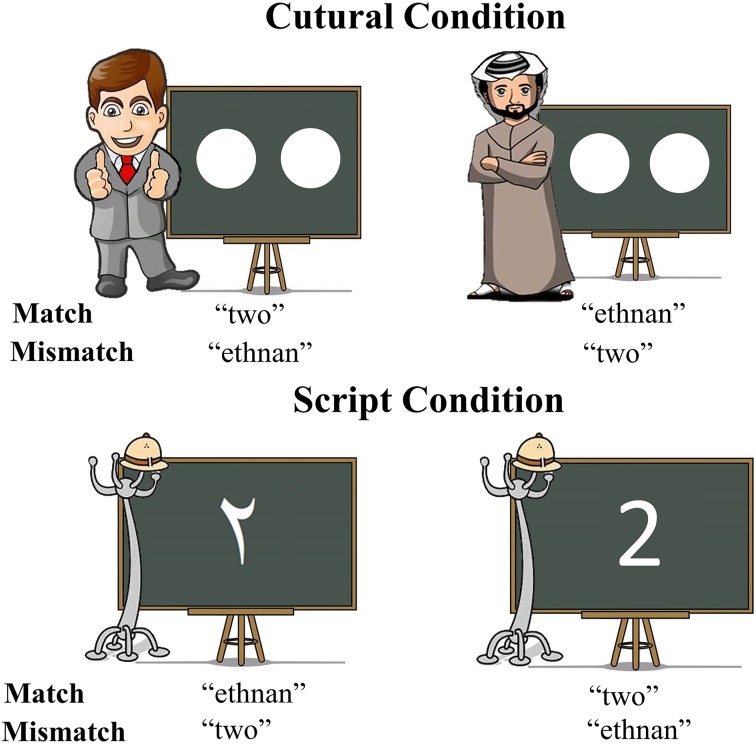
**Graphic depiction of all types of stimuli utilized in the experiment and correct responses for each of the experimental conditions**.

To achieve this, we selected two cues to language choice that are naturally employed in real life situations: script and cultural identity of the interlocutor. This specific contrast was chosen since the association between script and language is intuitively more direct and in some ways simpler than the association between cultural identity and language, which could potentially be a more multi-layered cue. We hypothesized that a more automatic link between a cue and a language should result in faster and less effortful retrieval of the lexical item to be produced. In our script conditions, Arabic-English bilinguals named number characters either in Arabic or English depending on the script of the displayed character, whereas in the culture conditions, the numerosities of dot-arrays were named in a language matching the cultural identity of a typically Arabic or Western interlocutor included in the display. The displays were informally matched for visual complexity across cue types (Figure 1) and a separate control experiment tested whether any observed effects of cue type could have simply reflected differences between naming number characters vs. dot arrays (Experiment 2). Crucially, this control experiment was conducted on monolingual individuals to assure that differences in activity observed in Experiment 1, if any, were associated to language selection mechanisms.

Since no attempt has previously been made to characterize the effect of natural cues on language selection, our regions of interest (ROIs) consisted of areas that have previously been implicated for language selection and switching. Prior research has proposed a distributed network for bilingual language control (Gruber and Goschke, 2004; Wang et al., 2008), hypothesizing such control processes to arise from the interaction of multiple discrete systems contributing complementary functions (Abutalebi and Green, 2008). Nevertheless, there are four areas that have consistently been reported to be part of the selection and language switching mechanisms in bilinguals: the inferior parietal lobule, the basal ganglia, the prefrontal cortex and the anterior cingulate cortex (ACC) (Abutalebi and Green, 2007; Abutalebi et al., 2008). However, the inferior parietal lobule has only been considered part of bilingual language control in as much as it relates to the maintenance of representations and working memory. Therefore, since the goal of the current experiment was to examine the role of different cues in language switching and response selection and not in working memory mechanisms—the task was not designed to posit extra memory load in any of the experimental conditions—this area was excluded from our ROI selection. We also did not aim to examine the basal ganglia, given that the ability of MEG to detect such deep sources is limited and challenging to localize (Lin et al., 2006; Attal et al., 2007). The basal ganglia has, however, been found to play a major role in the inhibition of competing alternatives (Crosson, 1985) and inappropriate behaviors (Casey et al., 2001); as well as in the suppression of alternatives in the process of integrating syntactic and semantic information (Friederici et al., 2003). Since some accounts (Green, 1998; Meuter and Allport, 1999) propose that bilingual individuals rely on reactive inhibition (i.e., the inhibitory process occurring after the lexical items of the non-target language are activated from the semantic system) for language switching, the basal ganglia could exhibit effects of our manipulation, which should be kept in mind when examining our question with techniques better suited for measuring the basal ganglia.

The most frequently reported regions as highly relevant for language switching in bilinguals have, however, been the LIPC and the ACC, and thus these areas constituted our primary ROIs. The hemodynamic literature on this topic has repeatedly found the left prefrontal cortex to be involved in decision-making and response selection and inhibition (Abutalebi and Green, 2007); specifically engaged by the need to select among competing response alternatives (Thompson-Schill et al., 1997, 1999; Badre and Wagner, 2002; Bunge et al., 2002; Moss et al., 2005). In other words, it is considered to house a top-down bias mechanism that enables the processing of relevant representation in the presence of stronger, irrelevant ones (Dehaene and Changeux, 1991; Desimone and Duncan, 1995; Miller and Cohen, 2001). In bilingual lexical selection studies, the left inferior prefrontal cortex (LIPC) has been identified within the left prefrontal cortex (PFC) as the most relevant region for language switching and selection. Rodriguez-Fornells et al. (2002, 2005, 2006) suggested that activation in the LIPC was associated with inhibition processes to reduce conflict in bilingual individuals, with converging evidence provided by van Heuven et al. (2008), who also found increased activation in the LIPC when language conflict was present (i.e., lexical items from both lexicons were simultaneously active).

The ACC, on the other hand, has mainly been reported as responsible for attention, conflict monitoring and error detection (Carter et al., 1998, 1999; Botvinick et al., 2001; Braver et al., 2001; van Veen et al., 2001; Ridderinkhof et al., 2004; Weissman et al., 2005). In our experimental design, to robustly activate language selection mechanisms and to investigate how they would override natural cue-language associations, we had participants perform not only the previously described Match task, in which the language that matched the cue was to be chosen, but also a Mismatch task, in which participants were given the opposite instruction. This task allowed us to test a straightforward behavioral hypothesis. If as we expected, script constitutes a more automatic cue to language choice than culture, the responses should be faster for this condition in the match task. However, in the mismatch task, a more automatic association should be harder to overcome and thus, would result in delayed responses. In addition, we created a clear conflict between each pair of possible responses to each stimulus. As a consequence, we predicted differences in the ACC since classic conflict tasks such as Stroop (Stroop, 1935) and Simon tasks (Simon and Small, 1969) have also consistently reported differences in the dorsal ACC (Fan et al., 2003; Liu et al., 2004). In addition, similar tasks involving a reassignment of response to stimuli (i.e., “shifting task” as in Wager et al. (2004) have also previously engaged the ACC (MacDonald et al., 2000). Therefore, based on the previous literature and taking into consideration the specific design of our experiment which included a Mismatch task, we narrowed the ROIs associated to language selection to LIPC and ACC with the goal of determining how the use of different language cues affects the neural mechanism for language switching and selection located in these brain areas.

In addition to the language selection related ROIs, we also examined early activity in the visual cortex, including the extrastriate and striate cortices as well as the fusiform gyrus of both left and right hemispheres. Previous literature has reported the laterality of object recognition to vary by the content of the stimulus (Mishkin and Forgays, 1952; Hilliard, 1973; Marcel and Rajan, 1975; Klein et al., 1976; Dien, 2009), with left hemisphere dominance for letters strings (Cohen et al., 2000; Dehaene et al., 2002; Cohen and Dehaene, 2004; Flowers et al., 2004; Dehaene and Cohen, 2011) and numerical characters (Geffen et al., 1971) whereas faces primarily activate right lateral occipitotemporal cortex (Bentin et al., 1996; Kanwisher et al., 1997; Linkenkaer-Hansen et al., 1998; Streit et al., 2000; Tarr and Gauthier, 2000; Gauthier and Palmeri, 2002; Tarkiainen et al., 2002; Grill-Spector et al., 2004; Palmeri and Gauthier, 2004; Tanskanen et al., 2005; Bukach et al., 2006). Since our culture conditions involved the recognition of a face whereas script conditions involved recognizing number characters, hemispherical differences at the early visual responses could have been expected as a factor of the utilized cue. Crucially, our ROI analyses were complemented with uncorrected full brain analyses aimed at confirming the results of the ROI analysis and revealing any robust patterns outside our ROIs.

## Experiment 1

### Methods

#### Participants

Twenty right-handed, native Arabic speakers participated in the experiment (19 male, one female, 22.3 years average 4.2 sd). All were neurologically intact, with normal or corrected-to normal vision and all provided informed written consent. To gather information about language use and proficiency level, the participants completed a language background questionnaire (Marian et al., 2007). All were native speakers of Arabic with a medium knowledge of English (from 5 to 8 in a 1–10 scale). They all reported the age of acquisition of English to be at the beginning of their formal education (mean age of 5). They all came from Arabic families and lived in an Arabic dominant linguistic environment [average level of exposure to this language 70% (*SD* = 18.84)] but were currently enrolled in an English speaking educational institution (see Supplementary Material for a precise linguistic profile of all participants).

#### Stimuli and experimental design

Stimuli were numerical values from one to four, and were displayed in two distinct forms. As previously mentioned, we wanted our paradigm to provide participants with natural cue-target language of response associations, with the goal of creating a setting that would maximally resemble a plausible real situation. In consequence, we chose as cues two of the features most intrinsically related to language: script and identity of the interlocutor. In the Script condition, numbers were presented in written form for participants to name and the script itself was the cue indicating the response participants had to provide. If numbers were presented in Arabic script, participants were instructed to produce their utterances in Arabic. Similarly, when participants were presented with a number in the Roman script, they were instructed to provide a response in English. The critical stimuli in the Culture condition were a number of white dots (from 1 to 4) drawn on a blackboard. The blackboard always had a prototypically Arabic man or a prototypically American (western) man next to it. This picture of the man was the indicator of which should be the language of response. In order to select which pictures best illustrated an absolutely obvious Arabic or American/British man that could be interpreted as their interlocutor, five pairs of possible Arabic/English prototypical depictions were created. An online poll was then opened and both American and British nationals and United Arab Emirates nationals voted for the pair that they considered represented their country and the other country best. Thirty people took part and the chosen pair was selected by 86.6% of the votes.

The number of dots in the blackboard only varied from 1 to 4 as previous studies (Saltzman and Gamer, 1948; Kaufman et al., 1949) have reported four to be the upper bound for the response to be subitized. By respecting this limit we tentatively assured that the response was automatic and no extra process of thought was required for participants to count the dots (Mandler and Shebo, 1982; Trick and Pylyshyn, 1994; Feigenson et al., 2004). In addition, the dots were displayed mirroring a dice to facilitate the recognition and stimuli for both conditions were matched for visual complexity. Therefore, although the display of the numbers to name varied, both conditions were essentially parallel number naming tasks. This claim is also supported by the fact that previous research has found that symbolic numerals (e.g., digits) and non-symbolic displays (dot patterns) follow the same identification process and are processed by overlapping brain regions (Piazza et al., 2007).

As previously mentioned, in addition to the Match task in which participants had to follow natural associations between cue and language, participants were also asked to perform a Mismatch task, in which they were asked to produce a response in the opposite language of what would be the natural way (Figure 1 for a graphic depiction of the experimental conditions).

The experiment consisted of a total of 384 trials. Half of the trials had Culture as a cue and the other half used Script for the same purpose. In addition, half of the items for each cue were presented in the Match task and half of them in the Mismatch task. Therefore, there were four experimental conditions containing 96 items each.

Furthermore, all experimental conditions encompassed the same amount of items to be produced in each of the languages at play and the same amount of repetitions for each numerical value. This allowed us to keep the motor production constant across all experimental conditions. In addition, it ensured that the same amount of switch and non-switch trials were included in all conditions, guaranteeing that no extra processing due to switching performance should be observed in any of them. Each experimental condition was further divided into two blocks of 48 to form the experimental blocks. Both items within blocks and blocks along the experiment were pseudo randomized following two constraints: two trials containing the same numerical value were never presented consecutively and two blocks of the same experimental condition never appeared successively. Participants were indicated at the beginning of each block whether the upcoming block was going to be a Match or a Mismatch block. All pictures were presented foveally using Presentation (Neurobehavioral System Inc., California, USA) and subtended in a range from 1.65° height and 2.55° width on a screen ~85 cm from the subject. The size of the picture and distance between the elements ensured that only one fixation was required to perceive all the elements of the stimuli, which was crucial to avoid saccade related artifacts.

#### Procedure

**Figure 2:**
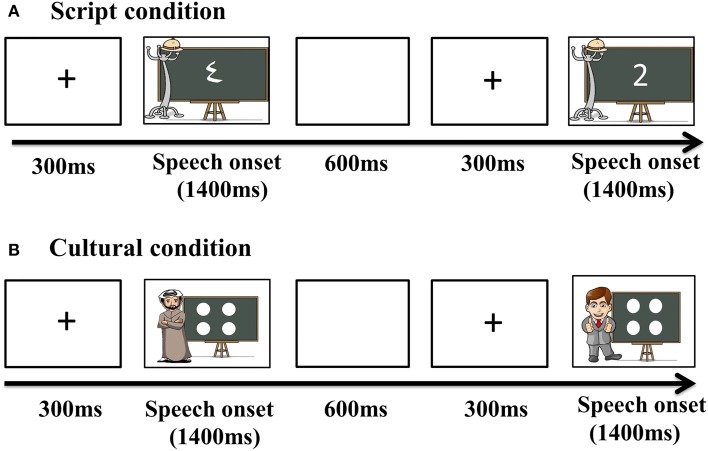
**Trial structure for the presentation of the stimuli**.

Before recording, each subject's head shape was digitized using a Polhemus dual source handheld FastSCAN laser scanner (Polhemus, VT, USA). MEG data were collected in the Neuroscience of Language Lab in NYU Abu Dhabi using a whole-head 208 channel axial gradiometer system (Kanazawa Institute of Technology, Kanazawa, Japan) as subjects lay in a dimly lit, magnetically shielded room. Vocal responses were captured with an MEG compatible microphone (Shure PG 81, Shure Europe GmbH). In all conditions, trials began with a fixation cross (300 ms), followed by the presentation of the stimuli. Pictures remained onscreen until speech onset began and participants were allowed 1400 ms to respond. After response, participants were given 600 ms to finish speech before the next trial began (Figure 2).

#### Data acquisition and preprocessing

MEG data were recorded at 1000 Hz (200 Hz low-pass filter) and epoched from 200 ms before to 700 ms after picture onset. For artifact rejection, a cut-off of 3000 fT was used for all participants except for one who showed a larger general amplitude range from the others. A cut-off of 3500 fT was used for this subject. Trials containing blinks were rejected after being identified by manual inspection of the MEG data. Altogether, this resulted in the exclusion of 27.3% of the trials (15.61% sd), leaving 280.7 trials on average per subject (59.94 sd).

The MEG data was noise reduced via the Continuously Adjusted Least-Squares Method (Adachi et al., 2001), in the MEG Laboratory software (Yokogawa Electric Corporation and Eagle Technology Corporation, Tokyo, Japan). Cortically constrained minimum-norm estimates (Hämäläinen and Ilmoniemi, 1994) were calculated via MNE (MGH/HMS/MIT Athinoula A. Martinos Center for Biomedical Imaging, Charleston, MA). The cortical surfaces were constructed by mapping an average brain from FreeSurfer (CorTechs Labs Inc., La Jolla, CA and MGH/HMS/MIT Athinoula A. Martinos Center for Biomedical Imaging, Charleston, MA) to the head-shape data gathered from the head-scanning process. This generated a source space of 5124 points for each reconstructed surface, and then, boundary-element model (BEM) method was employed to calculate the forward solution. Epochs were baseline corrected with the pre-target interval [−200, 0 ms] and low pass filtered at 40 Hz. Using the grand average of all trials for a particular subject, the inverse solution was computed from the forward solution. This determined the most likely distribution of neural activity. The resulting minimum norm estimates of neural activity were transformed into noise-normalized dynamic statistical parameter maps (dSPMs; see Dale et al., 2000; Sharon et al., 2007).

#### Data analysis

For each trial, the experimenter evaluated the participants' vocal responses and reaction times corresponding to incorrect responses were excluded from subsequent analyses. The following types of responses were scored as errors: verbal disfluencies (i.e., utterance repairs, stuttering), non-responses and responses in which participants produced a different name from that designated by the experimenter. Naming latencies below or above 2.5 SD from the mean were also discarded from the analysis. RTs were averaged over trials per condition and per participant and subjected to a 2 × 2 × 2 repeated measures analyses of variance (ANOVAs). Planned contrasts in Experiment 1 were also examined with paired *t*-tests (two tailed).

For the ROI analyses, following previous studies (Thompson-Schill et al., 1998; Poldrack et al., 1999; Wagner et al., 2000, 2001; Metzler, 2001; Köhler et al., 2004; Noppeney and Price, 2004; Grindrod et al., 2008; Heim et al., 2009), we included the posterior and dorsal extent [Broadmann Area (BA) 44] and anterior and ventral extent (BA45–47) of the left ventrolateral prefrontal cortex in the LIPC analysis. Following Devinsky et al. (1995), Paus (2001), and Ridderinkhof et al. (2004), we included in the ACC Broadmann areas 24 and 32 (referred to as “anterior” and “mid” cingulate, respectively in the Automated Anatomical Labeling (AAL) template [(Tzourio-Mazoyer et al., 2002), and the pregenual anterior cingulate cortex (BA 33)]. Lastly, for the analysis of the early responses in the visual cortex we included the extrastriate (BA18 and 19) and striate (BA17) cortices as well as the fusiform gyrus BA37 (Brodmann, 1903; Courtney et al., 1997; Flowers et al., 2004) bilaterally.

In order to assign each source to a Brodmann area, the Talairach Atlas Daemon was used (http://imaging.mrc-cbu.cam.ac.uk/imaging/MniTalairach). Following this atlas, each point in the cortex was automatically assigned to the nearest labeled Brodmann area (BA) and each point in the cerebellum to the nearest gyrus. This assignment of sources to BAs was then compared to an annotation file (lh.aparc.a2009s.annot) from Fs average data of FreeSurfer (CorTechs Labs Inc., La Jolla, CA and MGH/HMS/MIT Athinoula A. Martinos Center for Biomedical Imaging, Charleston, MA). If there was a discrepancy between the two, the sources were manually reassigned following the latter model to a different Brodmann area. For each of the Brodmann areas and gyri found, an MNE label was created, which used all the sources with which it was labeled as its vertices. In these areas, the noise-normalized dynamic statistical parameter maps (dSPMs; see Dale et al., 2000) resulting from the preprocessing of our data were submitted to a non-parametric permutation test which controls for multiple comparisons across the entire analysis window (Maris and Oostenveld, 2007) to identify temporal clusters with significantly different activation between conditions. The criteria for selecting clusters to be evaluated in this test is set beforehand (for more details see Maris and Oostenveld, 2007) and we selected 10 adjacent time points with *p* < 0.3 threshold following the parameters of prior studies (Bemis and Pylkkänen, 2011, 2012, 2013; Del Prato and Pylkkänen, 2014; Leiken and Pylkkänen, 2014; Pylkkänen et al., 2014; Westerlund and Pylkkänen, 2014) and enabling us to capture potential long-lasting effects in the data. The permutation tests were conducted within a very early time-window (50–200 ms) for the visual cortex ROIs (BAs 17, 18, 19, 37 bilaterally) and in two mid-latency time-windows (150–300 and 300–400 ms) for the ACC (BAs 24, 32, 33 bilaterally) and LIPC ROIs (left BAs 44, 45, 47). These intervals were motivated by the aim to capture early visual and language-cue processing effects as well as subsequent lexical selection activity, while excluding late activity reflecting any aspect of motor planning and/or execution, including associated artifacts. Within these intervals, a test statistic was defined for each observed cluster. This statistic was equal to the summed *t*-values of the point-by-point test-statistics over the selected cluster interval. Lastly, the data were subjected to random permutations and the final corrected *p*-value of the observed data was calculated as the ratio of permutations yielding a test statistic greater than the actual observed test statistic. Ten thousand permutations were used for all our analyses and to determine the significance of this final value, the standard alpha-level of *p* < 0.05 was used. At the end of the analysis, only waveform separations which fulfilled all the previous premises and where differences between conditions were sustained for a determined amount of time at our set alpha level were considered reliable. Cluster *p*-values were corrected for multiple comparisons across all BAs entered into a single analysis (i.e., separately for visual, ACC and LIPC regions) using the False Discovery Rate with an alpha level of 0.05 (Benjamini and Yekutieli, 2001; Genovese et al., 2002).

In addition, an uncorrected full brain analysis was performed in order to validate that the effects observed in the ROI analyses were indeed reflective of activity emanating from our ROIs and not vestiges from neighboring regions. Furthermore, this analysis also allowed us to detect possible effects localized outside of our ROIs. In this full-brain analysis, activity values were compared at every time-space data point using a paired samples *t*-test. The problem of multiple comparisons was alleviated by only considering effects significant if they remained reliable (*p* < 0.05) for at least 15 ms and 15 adjacent cortical sources.

### Results

#### Behavioral results

Three main variables were considered in the statistical analyses: Cue (Culture or Script), Task (Match or Mismatch) and Language of the stimuli (English or Arabic). Error rates and naming latencies were submitted to analyses of variance (ANOVAs) in a 2 × 2 × 2 design. Following the previously mentioned exclusion criteria 12.67% of the data were excluded directly from the analyses of the critical conditions (see Table 1 for distribution of errors and reaction times per condition).

**Table 1:** **Mean reaction times and percentage of errors across all conditions**.

**Condition**	**Correct RT (ms)**	**Errors (%)**
Cultural match	783 (180)	9.16 (7.42)
Cultural mismatch	879 (226)	12.65 (8.5)
Script match	744 (180)	11.19 (6.13)
Script mismatch	925 (237)	17.76 (11.06)

*Standard deviations are displayed in parentheses*.

**Figure 3:**
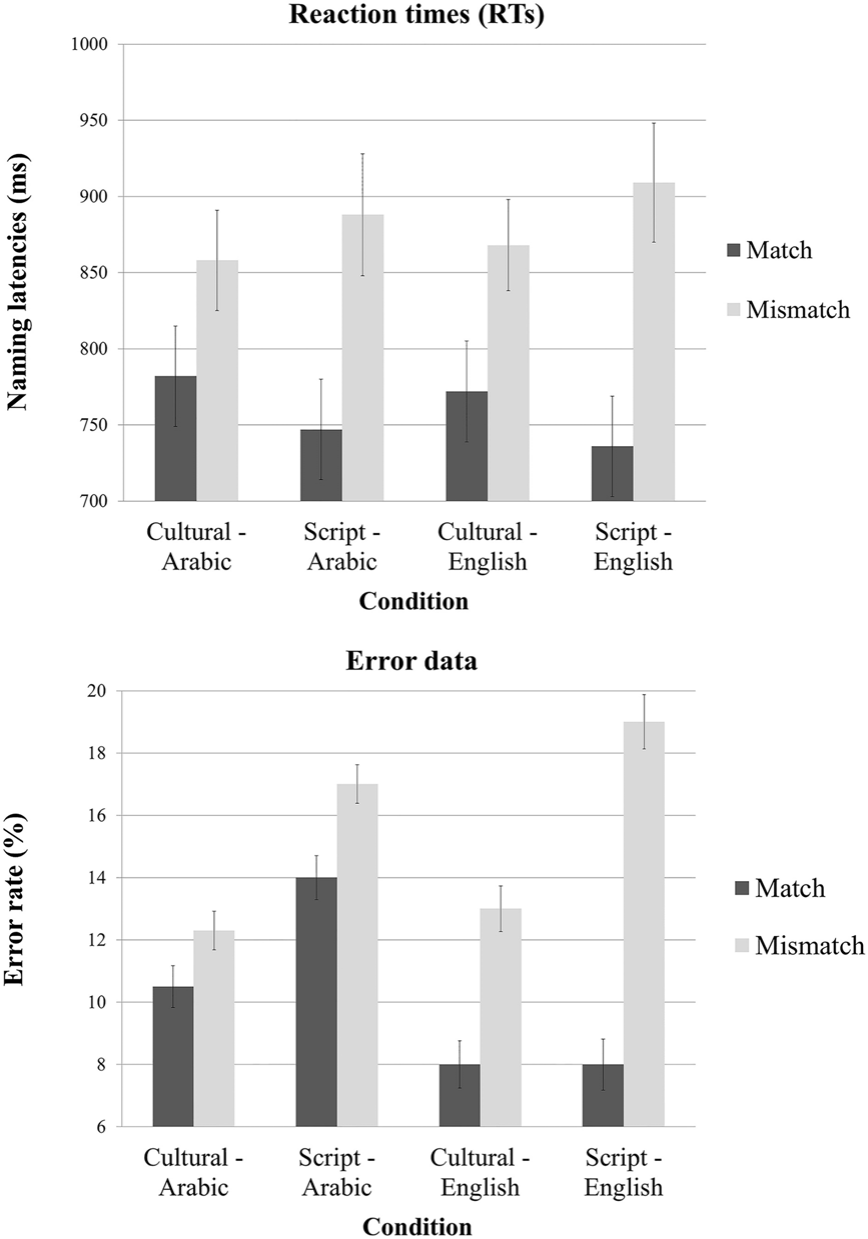
**Mean reaction times and error rate as a function of task, cue type and language of the stimuli**.

The 2 × 2 × 2 ANOVA on response time (RT) data elicited by our experimental conditions revealed a significant main effect of Task [*F*_(1, 13)_ = 245.5; *p* < 0.001] but not of Cue or Language. The interaction between Task and Cue was very significant [*F*_(1, 16)_ = 20.2; *p* < 0.0003] and the interaction between Task and Language was significant as well [*F*_(1, 17)_ = 6.3; *p* = 0.02]. Planned paired two tailed *t*-tests revealed that the Mismatch task yielded significantly slower reaction times both when the cue was Culture [*t*_(39)_ = 10.65; *p* < 0.001] and when the cue was the Script [*t*_(39)_ = 15.29; *p* < 0.0001]. Similarly, we tried to unpack the interaction between Task and Language and the pair-wise *t*-tests showed that there is a significant effect of Task type both for English [*t*_(39)_ = 11.52; *p* < 0.001] and for Arabic stimuli [*t*_(39)_ = 11.10; *p* < 0.001] (Figure 3).

The 2 × 2 × 2 ANOVA on error rate data elicited by our experimental conditions revealed a significant main effect of Task [*F*_(1, 13)_ = 24.64; *p* < 0.001] and of Cue [*F*_(1, 14)_ = 5.44; *p* = 0.03] but no main effect of Language. There was a significant interaction between Task and Language [*F*_(1, 17)_ = 6.49; *p* < 0.02] but no other interaction was reliable (however, the interaction between Task, Cue and Language was close to significant [*F*_(1, 19)_ = 3.61; *p* < 0.07]. Planned paired two tailed *t*-tests revealed that mismatch task yielded significantly more errors irrespective of the utilized cue {Culture [*t*_(39)_ = 2.52; *p* < 0.01] and Script [*t*_(39)_ = 4.22; *p* < 0.001]}. Similarly, we tried to unpack the interaction between Task and Language and the pair-wise *t*-tests showed that there is a significant effect of task type for English [*t*_(39)_ = 4.87; *p* < 0.001] and close to significant for Arabic stimuli [*t*_(39)_ = 1.91; *p* = 0.06] (Figure 3). Though the RTs and error rates for Cultural match and Script match trended toward a speed accuracy trade-off (Table 1), there in fact was no reliable different between the error rates of these two conditions [*t*_(19)_ = 1.21; *p* = 0.23].

#### ROI results

An ROI analysis was performed in the LIPC (Broadmann Areas 44, 45, and 47), ACC (Broadmann Areas 24, 32, and 33) and the visual cortex and fusiform gyri (BA 17, 18, 19, and 37). While the potential effects on the inferior prefrontal cortex were expected to be observed in the left hemisphere, we did not have specific laterality predictions for the ACC, therefore both hemispheres were included in the ACC analysis. Since the goal of the visual cortex analysis was to observe potential hemispheric differences, both hemispheres were included in this analysis as well.

**Figure 4:**
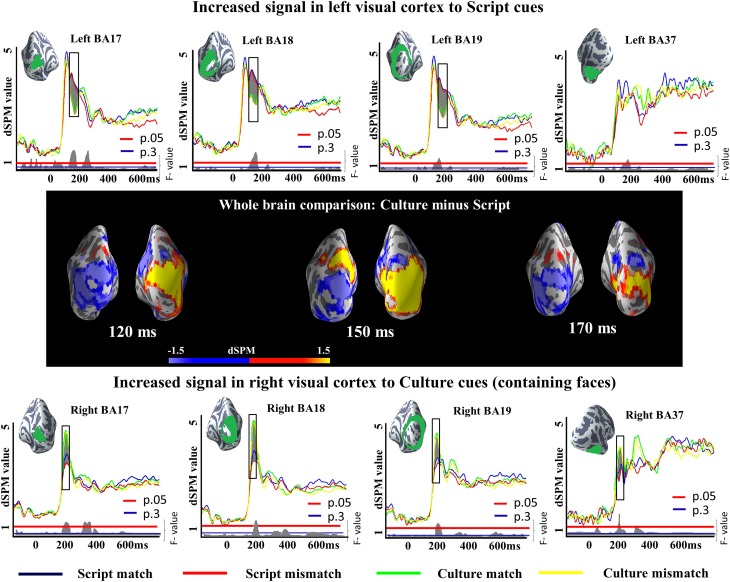
**ROI results for a 2×2 ANOVA in the visual cortex and fusiform gyrus, activation averaged across subjects**. On the waveform plots, the shaded regions indicate significant differences in activation as a factor of the utilized cue. In the left hemisphere, greater activity was observed when Script was utilized as a cue in comparison to when Culture was used for the same purpose. The opposite was true for the right hemisphere. Significance was determined using a non-parametric, permutation test (Maris and Oostenveld, 2007) performed from 50 to 200 ms (10,000 permutations). The whole brain comparisons show the subtraction of the activity elicited by Script cued conditions from the activity elicited by Culture cued conditions. In consequence, blue reflects increased activity for Script while yellow reflects increased activity for Culture.

We run a 2 × 2 analysis of variance (ANOVA) with Cue (Script or Culture) and Task (Match or Mismatch) as main factors in very early (50:200 ms), early (150:300 ms) and late (300:400 ms) time windows. The results revealed a reliable main effect of Cue in both of the earlier time windows and a reliable effect of Task in the later time window. The main effect of Cue in the earliest time window was observed in left BA17 (145–196 ms; *p* = 0.004), left BA18 (142–200 ms; *p* = 0.004), left BA19 (142–200 ms; *p* = 0.01), right BA17 (99–144 ms; *p* = 0.02), right BA18 (99–147 ms; *p* = 0.002), and right BA37 (97–168 ms; *p* = 0.0006). In addition, differences in right BA19 were also close to reliable (105–143 ms; *p* = 0.06). Importantly, while in the right hemisphere Culture conditions elicited greater activity than Script conditions, the opposite was true in the left hemisphere (Figure 4), conforming to prior literature on letter/character vs. face processing. We ran additional two-tailed *t*-tests between Culture and Script conditions within each of the tasks to unpack whether the differences observed in the ANOVA were reliable across tasks. The results of the *t*-test between Culture match and Script match revealed reliable differences in left BA17 (147–197 ms; *p* = 0.01), left BA18 (143–200 ms; *p* = 0.0009), left BA19 (144–200 ms; *p* = 0.02) and right BA37 (97–152 ms; *p* = 0.007), and a close to reliable difference in right BA18 (103–145 ms; *p* = 0.06). The pairwise comparison between Culture and Script within mismatch task revealed reliable differences in left BA17 (144–196 ms; *p* = 0.03), left BA18 (142–200 ms; *p* = 0.005), left BA19 (142–200 ms; *p* = 0.03), right BA17 (94–147 ms; *p* = 0.02), right BA18 (96–148 ms; *p* = 0.003) and right BA37 (102–146 ms; *p* = 0.009). Differences were also close to reliable in right BA19 (103–143 ms; *p* = 0.06). In both cases, Culture trials elicited greater activity in the right hemisphere and Script trials in the left hemisphere.

The main effect of Cue in the ACC extended to the three regions included in the analysis bilaterally. In the left hemisphere, reliable clusters of activation were found in BA24 (183–260 ms; *p* < 0.0001), BA32 (163–269 ms; *p* < 0.0001) and BA33 (163–270 ms; *p* < 0.0001) whereas in the right hemisphere the clusters were found from 150 to 252 ms (*p* = 0.0008) in BA24, from 150 to 252 (*p* = 0.001) in BA32 and from 194 to 249 (*p* = 0.002) in BA33. There was no significant interaction between Cue and Task and no cluster of activation was found in the ACC for the later time window in any of the analyzed areas.

No effect of Cue was found in the LIPC, however, a main effect of Task was found in BA45 and BA47 in the later time window. In BA44, the difference in activity between conditions did not reach significance (*p* = 0.09) although a cluster extending from 322 to 390 milliseconds was identified. In BA45 and 47 the Task effect was found reliable (313–400 ms; *p* = 0.01) and (309–400 ms; *p* = 0.006), respectively. No main effect of Cue was found in the later time window (BA44 *p* = 0.14; BA47 *p* = 0.7; no cluster found in BA45) and there was no interaction between factors (BA44 *p* = 0.4; BA45 *p* = 0.7; BA47, no clusters found).

**Figure 5:**
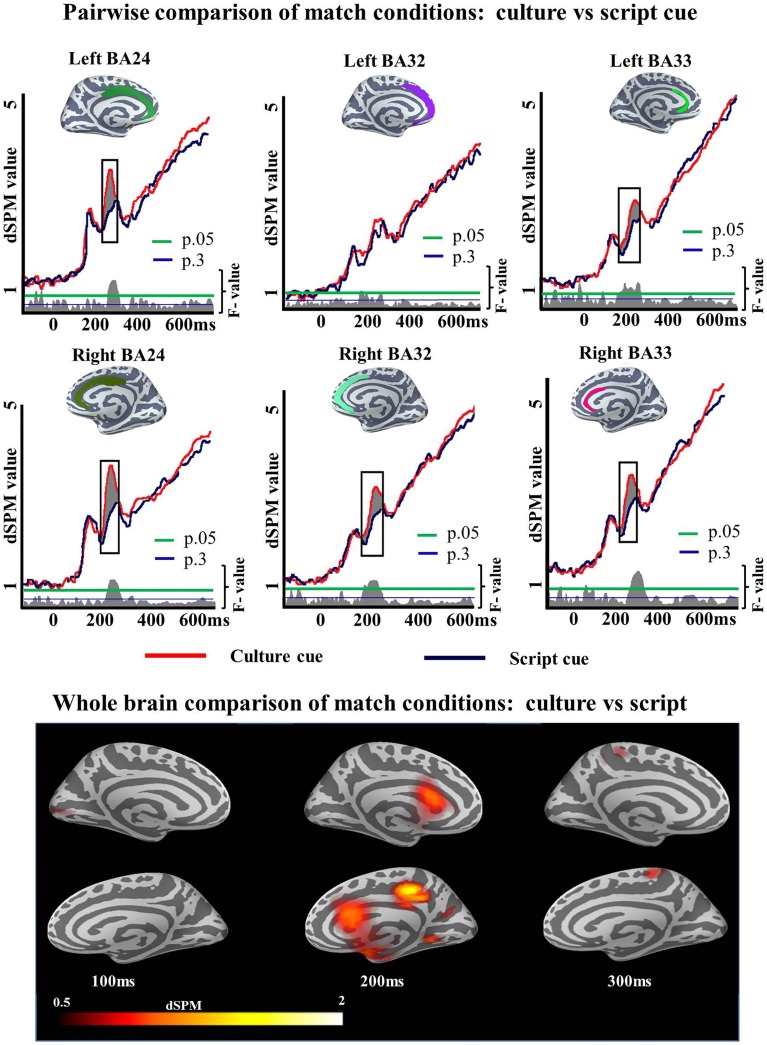
**ROI results for pairwise comparisons in the ACC, activation averaged across subjects**. On the waveform plots, the shaded regions indicate significantly greater activity when culture was utilized as a cue in comparison to when Script was used in the Match task. Significance was determined using a non-parametric, permutation test (Maris and Oostenveld, 2007) performed from 150 to 300 ms (10,000 permutations).

**Figure 6:**
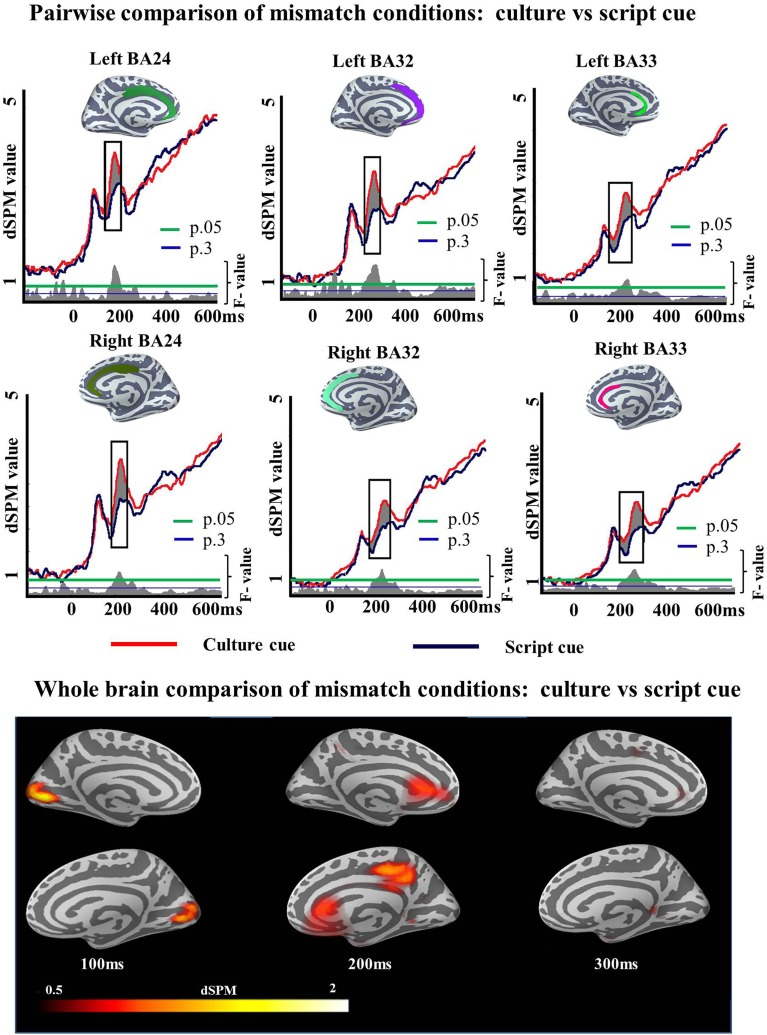
**ROI results for pairwise comparisons in the ACC, activation averaged across subjects**. On the waveform plots, the shaded regions indicate significantly greater activity when culture was utilized as a cue in comparison to when Script was used in the Mismatch task. Significance was determined using a non-parametric, permutation test (Maris and Oostenveld, 2007) performed from 150 to 300 ms (10,000 permutations).

To unpack the results of this ANOVA regarding language selection mechanisms we then examined the specifics of the Cue effect within each task and Mismatch effect within each cue. First we ran pairwise comparisons to determine whether the Cue effect of the ANOVA in the ACC was driven by either the Match or the Mismatch task. We ran two-tailed *t*-tests as we did not have any *a priori* prediction for increased activity for any of the cues. Within Match task, there was a reliable Cue effect in left BA24 (194–248 ms; *p* = 0.05), left BA33 (150–249 ms; *p* = 0.01), right BA24 (184–257 ms; *p* = 0.02), right BA32 (167–262 ms; *p* = 0.05), and right BA33 (174–255 ms; *p* = 0.04) but not in left BA32 (150–184 ms; *p* = 0.1) (Figure 5). Within Mismatch task the effects were more highly reliable overall. Significant differences between Culture and Script cued conditions were found in all analyzed regions of the left hemisphere: BA24 (188–274 ms; *p* = 0.008), BA32 (161–300 ms; *p* = 0.001), and BA33 (150–300; *p* = 0.0008), as well as in the analyzed areas of the right hemisphere: BA24 (162–300 ms; *p* = 0.0001), BA32 (154–300 ms; *p* = 0.0001), and BA33 (154–300 ms; *p* = 0) (Figure 6).

**Figure 7:**
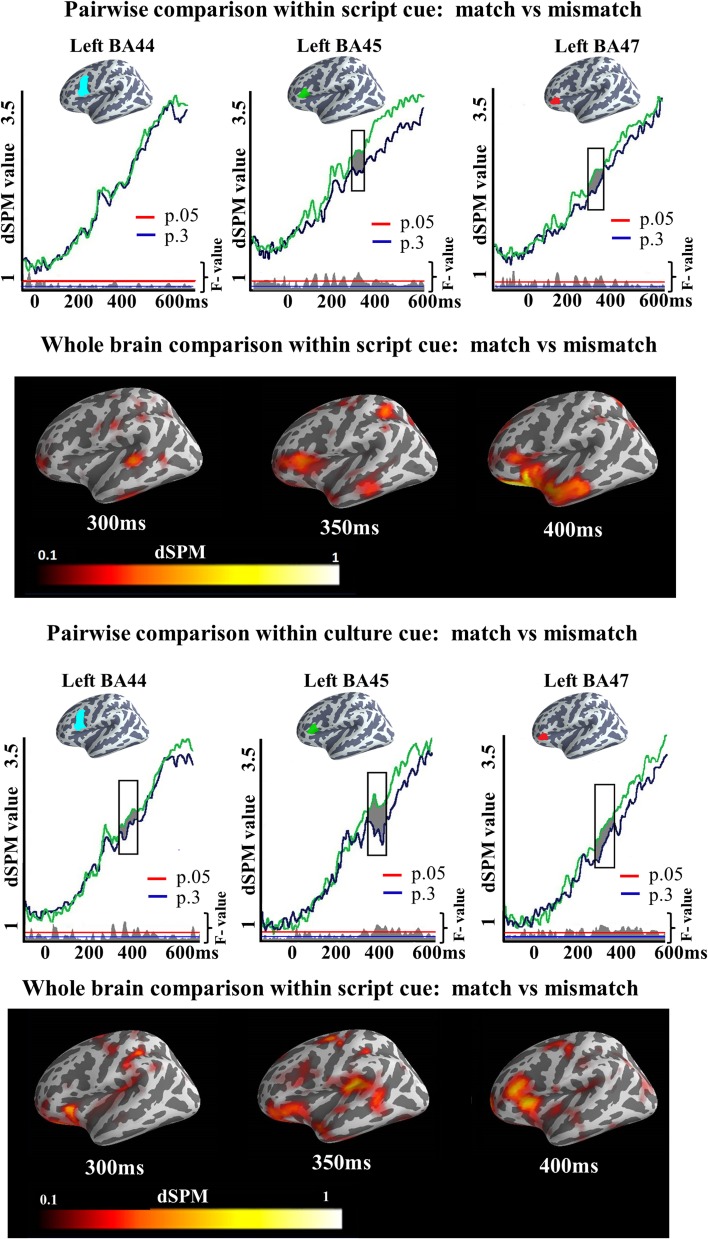
**ROI results for pairwise comparisons in the LIPC, activation averaged across subjects**. On the top, the locations of the ROIs are indicated in blue, green and red. On the waveform plots, the shaded regions indicate significantly greater activity when there was a mismatch in the association between cue and target language. Significance was determined using a non-parametric, permutation test (Maris and Oostenveld, 2007) performed from 300 to 400 ms (10,000 permutations).

We also ran two-tailed *t*-tests in both Culture and Script conditions in the later time window (300–400 ms) to assess the degree to which the increased activation found by the ANOVA for the Mismatch task differed depending upon the cue participants were presented with. In all the analyzed regions, clusters of activation showed increased activity elicited by the Mismatch as compared to the Match condition. In the Script condition, we observed a significant cluster of activation in left BA45 (317–389 ms; *p* = 0.03) and in left BA47 (319–371 ms; *p* = 0.04). No cluster was found in BA44. Within Culture condition, reliable clusters of activation were found in the three areas. In BA44 the cluster extended from 315 to 357 ms (*p* = 0.03), in BA45 from 365 to 400 ms (*p* = 0.03) whereas in BA47 the cluster extended from 300 to 380 ms (*p* = 0.01). Again, no area was found in which Match elicited more activation than Mismatch condition (Figure 7). It is worth mentioning that the dSPM statistic values for the clusters in which reliable increased activation was found for one of the experimental conditions were above the two dSPM value threshold. This assures when using dSPM methods that the observed results are due to activity elicited by the experimental conditions and not a consequence of overall noise [remember that the current estimate at each cortical location is normalized to the estimated baseline noise variance. Therefore, the output is given as a signal-to-noise ratio estimate and values above two mean that the activity is more than two standard deviations above the mean of noise (see Dale et al., 2000; Sharon et al., 2007)]. We did not exclude trials with response times faster than 700 ms (the end of our epoch), as we expected such trials to be rejected by our artifact rejection routine and the analyzed time windows were earlier than the speech onset time.

### Discussion

In this experiment, we aimed to examine the neurophysiological and behavioral effects of two different natural cues to language choice in bilingual language production: script and cultural context. We hypothesized that script cues language choice more directly than cultural context, and therefore, these two cues might affect language selection related activity differently. Our analyses focused on testing whether the ACC and the LIPC, previously implicated for bilingual language selection in production tasks, would also be engaged when using natural cues to language choice and whether they would show sensitivity to differences in the properties of these cues. Additionally, we aimed to explore whether these areas would be also influenced by the need to override natural cue-language associations and whether the particular properties of our cues, which required processing a face in culture condition and recognizing a numerical character in script conditions, caused hemispherical differences in early visual responses. Consistent with our hypothesis, both the visual cortex and the ACC showed reliable differences in activation as a factor of the utilized cue. Specifically, in the visual cortex at 50–200 ms, the effect pattern tracked the content of the visual stimulus in a predictable way, with the face-containing culture-trials eliciting increased right-lateral activation and the character-containing script-trials increased left-lateral activation. Shortly after the object-recognition related activity, the ROIs targeting activity related to language selection showed a robust increase in ACC activity for culture cued conditions compared to script cued conditions at 150–300 ms. In addition, increased activity was observed in the LIPC at 300–400 ms during the mismatch task in which natural associations needed to be overridden.

Although our stimulus choices succeeded in cueing language selection by means of two clearly ecological cues, there is an obvious difference between culture and script cued conditions: script conditions required naming a number character whereas culture conditions required naming the numerosity of dots. Thus, it is possible that the differences in activation found in the ACC were a consequence of this fact rather than a reflection of dissimilarities in the processing of the cues. To rule out this hypothesis, as well as to confirm that the detected difference was associated with language selection related activity, we conducted a second experiment assessing whether monolingual individuals would show similar effects if the cue was deleted and they performed a numerosity vs. number naming task. If the ACC results of Experiment 1 reflected language selection, Experiment 2 should yield a null result in the ACC. In contrast, if our ACC findings were driven by number character vs. dot naming, Experiment 2 should replicate them.

## Experiment 2

### Methods

Methods for this experiment were maximally parallel to Experiment 1 and thus only the methods that differed from Experiment 1 will be explained below.

#### Participants

Ten right-handed, native English speakers participated in the experiment (six male, four female, 25.9 years average 3.6 sd). Only 10 participants were tested in this control experiment as a test on the strength of the observed effects in Experiment 1 showed that this sample size was enough for them to be consistently reliable (see Section ROI Analysis). All were neurologically intact, with normal or corrected-to normal vision and all provided informed written consent. All participants reported English to be the only language in which they could communicate.

#### Stimuli and experimental design

**Figure 8:**
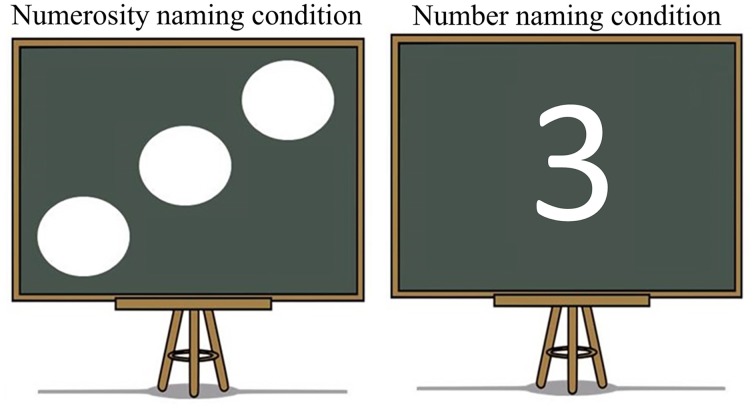
**Presentation of the stimuli for Experiment 2**.

In this experiment, the stimuli of the Culture condition of Experiment 1 were used but the cultural cues were deleted from them. Therefore, participants were presented with a blackboard which had one to four dots or the equivalent number drawn on it and they were asked to name the numbers in English as fast and as accurately as possible (Figure 8). The experiment consisted of a total of 192 trials. Half of the trials belonged to the Numerosity naming condition, in which participants had to name the number of dots on the screen, and the other half belonged to the Number naming condition, in which participants had to name the number presented onscreen. The experimental design was parallel to Experiment 1. There were two experimental conditions containing 96 items each and each of them was further divided into two blocks of 48 to form the experimental blocks. All experimental conditions encompassed the same number of repetitions for each numerical value to keep the motor production constant across both experimental conditions. Participants were told at the beginning of each block whether the upcoming block was going to be a Numerosity naming or a Number naming block. All pictures were presented foveally using Presentation (Neurobehavioral System Inc., California, USA) and subtended in a range from 1.65° height and 2.55° width on a screen ~85 cm from the subject.

#### Data acquisition and preprocessing

MEG data were recorded at 1000 Hz with a 200 Hz low-pass filter and epoched from 200 ms before to 500 ms after picture onset. We reduced the length of the epoch in comparison to Experiment 1 to avoid contamination of motion artifacts in our epochs, as behavioral reaction times were 200 ms faster on average (they began around 585 ms for the number condition). For artifact rejection, a cut-off of 3000 fT was used for all participants. Trials containing blinks were rejected after being identified by manual inspection of the MEG data. Altogether, this resulted in the exclusion of 14.2% of the trials (12.72% sd), leaving 165.7 trials on average per subject (24.42 sd).

#### Data analysis

For each trial, the experimenter evaluated the participants' vocal responses and reaction times corresponding to incorrect responses were excluded from subsequent analyses. The same criteria as in Experiment 1 were used to exclude behavioral responses. RTs from the remaining trials were averaged over trials per condition and per participant and subjected to a two-tailed *t*-test.

Since Experiment 2 was conducted strictly to address a possible explanation of the ACC effects in terms of differences in the naming task, only the time window (150–300 ms) and region (ACC) in which this effect was observed were analyzed. Mirroring Experiment 1 BA24, 32, and 33 were included in this ROI analysis. The noise-normalized dynamic statistical parameter maps (dSPMs) resultant from the preprocessing of our data were submitted to a permutation analysis, where the same cluster identification and selection criteria as in Experiment 1 were used. In addition, an uncorrected full brain analysis with identical parameters as those from Experiment 1 was performed.

### Results

#### Behavioral results

A two-tailed *t*-test was conducted on reaction time data for the two experimental conditions. A significant difference was found in terms of reaction times with participants being reliably faster in the number naming task (mean 535 ms) than in the dot naming task (mean 635 ms) (*t* = 8.9, *p* = 0.0008). Accuracy was at ceiling in both conditions, therefore, no analysis was run on it.

#### ROI analysis

**Figure 9:**
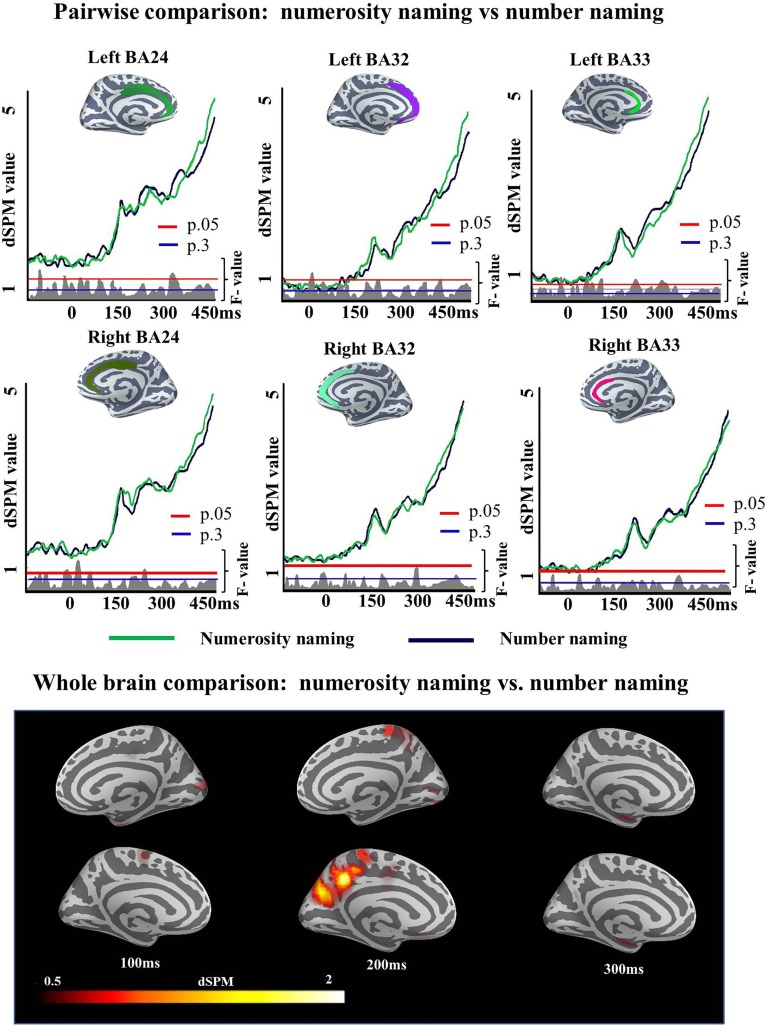
**ROI results for pairwise comparisons in the ACC between Numerosity and Number naming conditions in Experiment 2, activation averaged across subjects**. Significance was determined using a non-parametric, permutation test (Maris and Oostenveld, 2007) performed from 150 to 300 ms (10,000 permutations). No region showed reliable differences in activation between conditions in this analysis.

A single ROI analysis was run in the ACC. Since this experiment was designed as a control experiment for the Cue effects observed in Experiment 1, the areas and time window (150:300 ms) in which this effect was observed were included in the analysis. We tested the strength of the observed Cue effect in Experiment 1 (i.e., the number of subjects we needed to correctly accept the alternative hypothesis) and found that data for first 10 participants was sufficient for the effect to be reliable (left BA24 (175–258 ms; *p* = 0.02), left BA32 (150–256 ms; *p* = 0.008, left BA33 (150–259 ms; *p* = 0.005), right BA24 (176–300 ms; *p* = 0), right BA32 (159–300 ms; *p* = 0.02), and right BA33 (171–2300 ms; *p* = 0.003). A qualitatively similar pattern was obtained if we chose the last 10 participants or a random set of 10. As this power test in Experiment 1 had revealed 10 subject to be sufficient to reliably observe such an ACC effect, if it would exist, we ran a participant sample of this size in Experiment 2. The results of the two-tailed *t*-test showed that there was no significant difference between conditions in the monolingual population of Experiment 2 in any of the ROIs [left BA24 (193–213 ms; *p* = 0.62), left BA32 (241–269 ms; *p* = 0.45), left BA33 (232–268 ms; *p* = 0.36), right BA24 (176–188 ms; *p* = 0.76), right BA32 (238–256 ms; *p* = 0.52), right BA33 (235–255 ms; *p* = 0.66)], i.e., no increases was found for Numerosity naming condition over Number condition (Figure 9).

### Discussion

Experiment 2 was a targeted investigation aimed at ruling out an explanation of the findings of Experiment 1 based on the differences in the nature of the naming task (naming numerosities in culture condition vs. naming numbers in script condition). In addition, it also aimed to confirm that the observed effects were reflecting differences in language selection related activity. If the ACC increases for culture over script cued trials in Experiment 1 had been due to the requirement to name a numerosity in this condition, a similar increase should have been observed in the Numerosity naming condition of Experiment 2 (in comparison to the Number naming condition of the same experiment). In addition, monolingual individuals tested in Experiment 2 also showing increased ACC activity would have made our claim that the observed effect was a consequence of language switching performance unsustainable.

However, no significant difference was found in ACC activation between Numerosity and Number naming conditions. This result confirms that the observed Cue effects in Experiment 1 are not a consequence of differences in the naming task and suggests that differences in ACC activity in Experiment 1 are related to language selection.

## General discussion

In the present study, we examined the neurophysiological and behavioral effects of two different natural cues to language choice, script and cultural context. We hypothesized script to be a more automatic cue to language choice than cultural context, and that in consequence, these two cues might affect language selection related activity differently. In addition, a mismatch task addressed the mechanisms responsible for overriding natural cue-language associations. Our results revealed that as expected, early neural activity in the visual cortex was influenced by the content of the visual display, with script conditions eliciting greater activity in the left visual cortex and face-containing culture conditions in the right visual cortex. Temporally, these effects were observed around 170 ms after the presentation of the stimuli, consistent with previous studies on letter and face perception studies (Bentin et al., 1996; Linkenkaer-Hansen et al., 1998; Solomyak and Marantz, 2009). In the more anterior ROIs hypothesized to support language selection, our results revealed a strong increase for cultural cues in the ACC, an effect that was crucially absent when monolingual participants performed a maximally parallel task. In contrast, the LIPC showed a mismatch effect that was non-sensitive to cue type. In sum, our results implicate distinct regions of the executive control system as responsible for natural but less automatic language selection and for the inhibitory mechanisms that serve to overcome instinctive cue-language associations.

### The sensitivity of the anterior cingulate cortex to cue type

Results from Experiment 1 showed a robust sensitivity in the ACC to the utilized cue, with culture cued trials eliciting significantly greater activation than script cued trials during both match and mismatch tasks in an early time window. No such effect was observed for monolingual participants during a maximally parallel task in Experiment 2 and thus it is likely that the increase in ACC activity for culture cued trials indeed reflected greater executive control required when language selection is cued by culture.

The ACC modulation by cue type fits quite straightforwardly into prior literature on the ACC, specifically in relation to its role in attention (Barch et al., 1997; Orr and Weissman, 2009). Traditionally, attention has been divided into three networks (Posner and Petersen, 1990), carrying out the functions of alerting, orienting, and executive attention/control. Alerting is defined as achieving and maintaining an alert state; orienting is defined as the aligning of attention with a source of sensory input—and it is manipulated by presenting a cue indicating where in space a person should attend—(Posner, 1980); and executive control is defined as the effort to retrieve for example a word amongst competing responses (Posner and Rothbart, 2000). Several prior studies have observed increased ACC activity for greater executive control demands (Bush et al., 2000; MacDonald et al., 2000; Posner et al., 2006) and thus we hypothesize that the increased ACC activity elicited by culture cued trials may reflect the greater executive control required to retrieve the target element when presented with a culture cue as compared to a script cue. More specifically, the decreased demand on executive control for script would relate to less effortful retrieval, by hypothesis resulting from a more automatic and stronger association between a script cue and the target language. This stronger association is also reflected in the behavioral results. Firstly because faster responses were found for script condition in the match task, suggesting that in a natural processing situation, when the usual links between cue and language are respected, Script is the most efficient cue to language selection. Secondly because participants were significantly slower and more error prone in this condition during mismatch task, suggesting greater difficulty found in overcoming script-target language associations as compared to culture-target language associations, reflecting by hypothesis the stronger link between the two. Importantly, the ACC has previously been suggested as the locus of bilingual language control and language selection conflict resolution (Crinion et al., 2006; Wang et al., 2007, 2009; Abutalebi et al., 2011; Guo et al., 2011).

While Experiment 2 rules out an explanation of our ACC findings in terms of numerosity vs. number character naming, the script and culture cue conditions still differed in one way that could in principle have caused our ACC effect, namely, that culture conditions required participants to consider two elements (the dots and the person) in order to retrieve the appropriate item whereas in script conditions, a single element (the number character) conveyed all the relevant information for target word production. Following the definition of the attentional network described above (Posner, 1980), if considering one vs. two elements caused the Cue effect, it would have been as a consequence of differences in the requirements on the orienting network. Previous studies on orienting and visuospatial attention have reported areas distinct from the ACC—mainly the superior parietal lobule, the intraparietal sulcus, the frontal eye field, the supplementary eye field, the superior colliculus, the pulvinar, and reticular thalamic nuclei (Posner, 1980; Fink et al., 1997; Corbetta et al., 2000; Corbetta and Shulman, 2002; Fan et al., 2002; Xu et al., 2010) and the posterior cingulate cortex (Small et al., 2003) to be consistently activated as a consequence of changes in spatially directed attention. In addition, differences in visuospatial attention have been reported when subjects were asked to maintain fixation at a central fixation point and to direct attention covertly to peripheral target locations to detect a stimulus (Corbetta et al., 1993, 1998; Nobre et al., 1997; Gitelman et al., 1999; Kim et al., 1999), to discriminate it (Fink et al., 1997) or to track its movement (Culham et al., 1998) by means of visually demanding tasks such as different versions of the Flanker task (Eriksen and Eriksen, 1974). However, reportedly no study has found differences on the orienting network as a consequence of the type of visual variations in the stimuli that Experiment 1 presents. Therefore, in light of this literature, our ACC effect is unlikely to relate to the visuospatial differences in our stimuli and is more likely related to the higher level executive demands associated with language selection.

### LIPC mismatch effect

Mirroring the behavioral delay associated with mismatch trials, the analysis of the LIPC revealed a reliable activation increase at 300–400 ms for the mismatch conditions, where subjects were asked to select a language opposite to the cued one. The LIPC effect was significant in both BA45 and BA47, with a similar but less reliable pattern also observed in BA44. Our results conform to previous findings in bilingual language production studies in which the LIPC was found to be sensitive to selection demands and the level of conflict presented by the task (Rodriguez-Fornells et al., 2002, 2005, 2006), as well as response selection and inhibition (Abutalebi and Green, 2007). Specifically, our results can be straightforwardly predicted by Rodriguez-Fornells et al.'s (2002) proposal suggesting that activation in BA45 is be related to inhibition of the non-target language, given that activity in the LIPC in general and in BA45 in particular significantly increased when the selection difficulty and inhibition demands increased during our mismatch task.

However, there is also a body of evidence from monolingual lexical retrieval that has typically not been considered within the bilingualism field that could largely account for our LIPC effects. In opposition to the hypothesis that the LIPC plays a major role in lexical retrieval (Wagner et al., 2001; Badre and Wagner, 2002), a contrasting hypothesis has proposed that it is not retrieval of semantic knowledge *per se* that is associated with LIPC activity but rather selection of information among competing alternatives from semantic memory (Thompson-Schill et al., 1997). These accounts suggest that the LIPC plays a major role when automatic access is insufficient due to weak semantic cue target associations (Raichle et al., 1994; Gabrieli et al., 1998; Wagner et al., 2001), and this could explain our activation pattern. In our mismatch conditions where the cue was misguiding language choice, greater activity was found in the LIPC. This conforms to previous findings (Gabrieli et al., 1998; Raichle et al., 1994; Wagner et al., 2001) and suggests that demands on LIPC are proportional to the extent to which the target element can be retrieved through bottom-up cue-guided retrieval. Therefore, the increased activity in the LIPC during the mismatch task would be a reflection of the top-down inhibition demands posited onto it to allow the word belonging to the target language to be retrieved. The fact that increased activity for mismatch condition was found irrespective of the utilized cue to language choice suggests that this mechanism is associated with overriding the natural associations, irrespective of the strength of the natural association itself. This view is also consistent with one of the prevalent views on bilingual lexical access and selection (Inhibitory Control Model; Green, 1998) which suggests that inhibition needs to be applied to allow the target element to be retrieved in situations in which the equivalent of the other language will be the dominant one, which was the situation in our mismatch task.

### Lack of mismatch effect in the ACC

As already mentioned above, the ACC has been reported as sensitive to conflict monitoring (Carter et al., 1998; Botvinick et al., 2001; Braver et al., 2001; Swick and Turken, 2002; Ridderinkhof et al., 2004; Kern, 2006) in tasks such as Stroop (Stroop, 1935) or Simon (Simon and Small, 1969) tasks. Thus, since the mismatch task was essentially a Stroop-like task, one might have expected it to elicit greater activity in the ACC than the match task. However, no increase for mismatch conditions was observed in the data for Experiment 1.

One way to understand the lack of an ACC mismatch effect is in light of prior studies that have found stronger activations of the left PFC to associate with smaller interference effects in the ACC and stronger activation of the ACC with larger interference effects (MacDonald et al., 2000). Specifically, is has been proposed that individuals with good control mechanisms are better at maintaining top-down controlling representations in the PFC, and therefore need to draw less on the conflict resolution processes associated with the ACC (Jonides et al., 2002). Since bilinguals have been repeatedly reported to have improved top-down mechanisms and cognitive control (Bialystok et al., 2005; Abutalebi et al., 2011), this account would suggest that it is this bilingual advantage that might reduce the requirements on the ACC during mismatch tasks for this population in comparison to similar tasks in monolingual populations.

### Timing of language selection

The sum of our results provides two main novel aspects to the understanding of the neural basis of language selection. First, our results implicate the ACC for the processing of natural cues to language choice in an early time-window of 150–300 ms, suggesting that the processing of these cues may be achieved at around 200 ms after their presentation, following the initial cue recognition achieved at ~170 ms. In addition, the presence of this ACC effect in both the match and mismatch tasks suggests that there is a bigger cost derived from processing a cultural cue than a script cue irrespective of the task an individual is performing. However, the fact that these differences were stronger and wider-spread in the mismatch task suggests that the level of conflict associated with the target language of response also influenced the manner in which language cues were processed. Together, these findings suggest that not all natural cues cue language choice as effectively and in this case in particular, it reveals that although both ecologically valid, script constitutes a more automatic cue to language choice as compared to culture.

Second, our findings implicate the LIPC for a top-down bias mechanism allowing the retrieval of a lexical item in a non-prevalent language. Thus, overcoming the first intuitive response in bilinguals could be a somewhat similar process to overcoming the predominant response in monolinguals as described by previous monolingual lexical retrieval studies (Miller and Cohen, 2001). Since our LIPC effect was observed at a later time window (300–400 ms) than the Cue effect, we hypothesize that while the cue itself could be processed at a pre-lexical level, overriding natural cue-language associations may occur during lexical selection.

## Conclusion

Overall, this study demonstrates that regions emerging as lead candidates for language selection from prior literature appear to participate in language selection when natural cues are utilized. In addition, the difference in ACC activation observed between culture and script conditions together with the behavioral data suggest that not all language cues associate to a target language similarly. In this case, although both cues were ecologically valid, our results suggest that script is a more efficient cue to language choice than the identity of the interlocutor, as indexed by reduced executive control demands and faster reaction times for the former. Lastly, this study shows evidence for the role of the LIPC in top-down inhibitory mechanism for overriding natural cue-language associations.

### Conflict of interest statement

The authors declare that the research was conducted in the absence of any commercial or financial relationships that could be construed as a potential conflict of interest.
