# Supplementary material for: Brain bases of language selection: MEG evidence from Arabic-English bilingual language production
Source: Front Hum Neurosci. 2015 Feb 5;9:27. doi: 10.3389/fnhum.2015.00027 (PMC4318278; doi:10.3389/fnhum.2015.00027)
Supplement: Supplementary file 1 [file DataSheet1.PDF]

Supplementary Material 1. Participants' language background (Marian et al., 2007).

ID	% EXPOSITION L1	% EXPOSITION L2	LEVEL L1	LEVEL L2	% WRITE L1	% WRITE L2	%READ L1	%READ L2	% SPEAK L1	% SPEAK L2	% HEAR L1	% HEAR L2	SPEAKING L1	UNDERSTAND L1	WRITING L1	READING L1	SPEAKING L2	UNDERSTAND L2	WRITING L2	READING L2
**Participant 1**	35	65	9	7.75	40	60	20	80	30	70	25	75	10	9	8	9	7	8	8	8
**Participant 2**	30	70	10	8.5	30	70	25	75	30	70	30	70	10	10	10	10	7	9	9	9
**Participant 3**	80	20	7	6.25	90	10	30	70	90	10	80	20	8	8	6	6	5	7	7	6
**Participant 4**	85	15	9.75	6.75	50	50	70	30	80	20	70	30	10	10	9	10	6	7	7	7
**Participant 5**	85	15	9	7	40	60	70	30	90	10	80	20	9	9	8	10	6	7	6	9
**Participant 6**	70	30	10	6.5	75	25	60	40	80	20	50	50	10	10	10	10	6	6	7	7
**Participant 7**	55	45	9.25	6.5	80	20	25	75	60	40	60	40	10	10	8	9	7	7	6	6
**Participant 8**	50	50	8.5	7.25	10	90	20	80	70	30	75	25	9	9	8	8	7	7	7	8
**Participant 9**	90	10	10	6.75	90	10	75	25	50	50	90	10	10	10	10	10	6	6	6	9
**Participant 10**	45	55	10	6.75	30	70	25	75	30	70	30	70	10	10	10	10	5	7	7	8
**Participant 11**	55	45	9.75	7.25	10	90	30	70	60	40	40	60	10	10	9	10	7	7	7	8
**Participant 12**	80	20	10	7	80	20	40	60	90	10	80	20	10	10	10	10	6	6	7	9
**Participant 13**	80	20	9.5	5.5	60	40	30	70	20	80	25	75	10	10	9	9	5	6	5	6
**Participant 14**	60	40	8.75	6.75	50	50	45	55	75	25	60	40	9	9	8	9	7	7	6	7
**Participant 15**	80	20	9.75	6.25	45	55	50	50	70	30	65	35	9	10	10	10	6	6	6	7
**Participant 16**	90	10	9.5	5.25	70	30	35	65	80	20	50	50	10	10	9	9	5	5	5	6
**Participant 17**	85	15	9.5	6.5	50	50	55	45	75	25	70	30	10	10	8	10	6	6	7	7
**Participant 18**	75	25	9	6.5	30	70	60	40	70	30	70	30	9	9	9	9	6	7	6	7
**Participant 19**	85	15	10	5.75	60	40	60	40	80	20	80	20	10	10	10	10	5	6	6	6
**Participant 20**	85	15	10	5.75	40	60	45	55	65	35	70	30	10	10	10	10	5	7	6	5
**Average**	**70**	**30**	**9.41**	**6.62**	**51.5**	**48.5**	**43.5**	**56.5**	**64.75**	**35.25**	**60**	**40**	**9.65**	**9.65**	**8.95**	**9.4**	**6**	**6.7**	**6.55**	**7.25**
(sd)	***(18.84)***	***(18.84)***	***(0.74)***	***(0.74)***	***(23.95)***	***(23.95)***	***(17.99)***	***(17.99)***	***(21.79)***	***(21.79)***	***(20.51)***	***(20.51)***	***(0.58)***	***(0.58)***	***(1.09)***	***(0.99)***	***(0.79)***	***(0.86)***	***(0.94)***	***(1.20)***
